# Mesopic Illumination in Natural Environments: Implications for Myopia Research

**DOI:** 10.1007/s44402-026-00114-3

**Published:** 2026-07-02

**Authors:** Natalia Carolina Pereyra, Carla Lanca, Matias Acerbi, Guillermo Saracco, WeiZhong Lan, Rafael Iribarren, Jos J. Rozema

**Affiliations:** 1 Ophthalmology Department, Clinics University Hospital, Buenos Aires, Argentina; 2 Division of Science, New York University Abu Dhabi, Abu Dhabi, United Arab Emirates; 3 Comprehensive Health Research Center (CHRC), Escola Nacional de Saúde Pública, Universidade NOVA de Lisboa, Lisboa, Portugal; 4 Foucault Optical Laboratory, Buenos Aires, Argentina; 5 Saracco Optical Laboratory, Buenos Aires, Argentina; 6 Guangzhou Aier Eye Hospital, Jinan University, Guangzhou, China; 7 Novar, Miami, Florida USA; 8 Drs. Iribarren Eye Consultants, Buenos Aires, Argentina; 9 Retina Foundation, Buenos Aires, Argentina; 10 Institute for Medical Informatics, Statistics and Epidemiology (IMISE), Leipzig University, Leipzig, Germany; 11 Visual Optics Lab Antwerp (VOLANTIS), Faculty of Medicine and Health Sciences, Antwerp University, Antwerp, Belgium; 12 Department of Ophthalmology, Antwerp University Hospital, Edegem, Belgium

**Keywords:** Mesopic illumination, Natural environments, Outdoors

## Abstract

**Purpose:**

This study aims to determine the duration of mesopic light conditions in natural environments.

**Methods:**

Illuminance levels were measured at three outdoor locations (a city terrace, a forest and a shadowy park) during different seasons using a calibrated high-spec lux meter with a sensitivity threshold of 0.01 lux. Measurements were recorded from 1 h before sunset until complete darkness to capture the transition from photopic to mesopic (<40 lux) and scotopic (<0.1 lux) conditions. The duration and onset of mesopic light were analysed across locations and seasons to identify site-specific variations.

**Results:**

A gradual decline in illumination was seen from photopic light at 1 h before sunset down to mesopic and scotopic levels. Among the three measured locations, the shadowy park transitioned to mesopic conditions 15–20 min earlier than the terrace or forest. However, despite these differences in onset, the duration of the mesopic period remained consistent, lasting approximately 25–30 min across all locations during the different seasons.

**Conclusions:**

In natural outdoor environments, mesopic light exposure during sunset represents a relatively brief and consistent transition period of approximately 25–30 min. These findings provide objective field-based baseline data on the temporal dynamics of mesopic illumination. While the present study did not assess refractive development or myopia status, the quantified environmental parameters may inform future research integrating wearable light sensors and refractive outcomes.

Key Points

- Children spend increasing amounts of time under mesopic illumination conditions. The maximum natural exposure to this condition remains to be established.
- The natural mesopic exposure at sunset is 20 min at the equator, increasing to 30 min at latitudes of ±35°, and much longer beyond. It also varies throughout the year.
- This factor must be considered when comparing experiments monitoring light exposure.

## Introduction

Myopia has become a global health issue since the latter half of the twentieth century, but this was not the first recorded epidemic, as a similar occurrence was reported in Europe during the nineteenth century [1]. This epidemic was possibly mitigated by a public health movement that proposed exposure to open air and sunlight as the best way to prevent myopia and other diseases prevalent at that time, such as rickets, tuberculosis and other infections [2, 3]. Another example of the impact of myopic environmental risk factors is the acculturation of Inuit communities in the Arctic that led to a myopia epidemic in 1960–1970 [4]. And from then onwards, an epidemic of myopia began  in East and Southeast Asian urban environments, with China and other rapidly developing countries like Singapore, Korea and Japan developing high academic study programmes and affluent urban dwellings after 1980 [5–7]. These epidemics suggest an environmental causality for this shifting incidence and prevalence of myopia (as genes do not change in one generation) [8]. During these last epidemics, experimental models of myopia were developed that confirmed that the main cause of myopia was an increased rate of axial elongation [9]. In experimental models of fish, birds and mammals, including primates, it was discovered that the eye controlled the rate of growth by decoding the focus of the image at the retinal plane to adjust ocular dimensions to the focal distance of the cornea and crystalline lens [9, 10]. At a physiological and biochemical level, gene expression is implicated in the circuits that decode defocus at the retinal plane, thereafter sending a message across the choroid up to the sclera to modify its rate of elongation [10]. Although genes play a role, genetic differences in hundreds of genes only explain about 20% of the myopia phenotype, leaving the rest of the variability to environmental factors [11]. Thus, it is thought that environmental and lifestyle habits have driven the myopia epidemics in those highly developed urban environments [12].

A recent review concluded that higher incidence and progression of myopia are associated with children spending less time outdoors and more time in dim indoor light environments while being engaged in sustained near vision activities [13]. However, robust data specifically quantifying the independent role of dim (mesopic) indoor illumination remains limited. Another review by the International Myopia Institute about the role of light in refractive development highlighted the importance of outdoor exposure to sunlight in animal and clinical studies, but devoted limited attention to mesopic indoor illumination levels, largely due to the scarcity of robust data [14]. Since it was first described by Rose and Morgan in 2006 [15], evidence for the importance of the time spent outdoors has mounted, as outdoor exposure protects against myopia in children [16] and animal models [9, 17]. The evidence emphasises that not only is time spent outdoors important, but also the ambient light intensity [18]. The high luminance levels found outdoors could be the principal beneficial feature, possibly related to the increased dopaminergic activity in the retina [13]. This retinal dopamine release is stimulated by light exposure, and seminal studies in chick and monkey models demonstrated that dopamine turnover was activated by increased illumination, while experimental myopia was arrested [19]. Furthermore, dopamine agonists stimulate this protective effect, whereas dopamine antagonists lead to more myopic development.

While numerous studies have examined outdoor light exposure in relation to myopia, most have focused on photopic illumination levels, typically emphasising exposures above 1000 lux [20, 21]. Substantially less attention has been paid to illumination within the mesopic range. Interestingly, children who spend more time in mesopic light levels before sleep showed more myopia progression [22]. As demonstrated by Read et al. [23], most children stay in mesopic light environments after 17:00 h between school and bedtime (21:00 to 23:00 h) [23]. Hence, children tend to stay in mesopic light conditions for 4–6 h a day when living in modern buildings and houses. However, wearable light sensor studies often lack sufficient sensitivity at very low illumination levels, and existing meteorological records commonly exclude dawn and dusk transition periods. As a result, the natural duration of mesopic outdoor light exposure remains poorly characterised. Understanding how long mesopic illumination persists in natural environments is a necessary first step before evaluating its potential relevance to ocular growth and myopia development. Thus, the present study aims to quantify the duration of mesopic light conditions in natural outdoor environments.

## Materials and Methods

This work was designed as a field-based environmental measurement study aimed at quantifying the duration of mesopic illumination in natural outdoor settings. The objective was not to test a causal relationship with myopia, but to document real-world illumination conditions that are not captured in conventional meteorological datasets or most wearable light sensor studies.

**Fig. 1:**
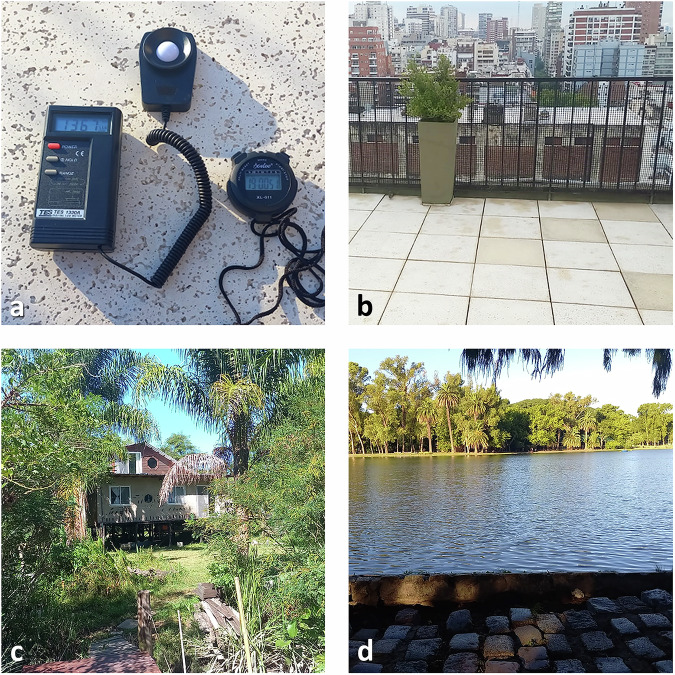
**a** Lux meter used for the measurements; **b** terrace location; **c** forest location; **d** park location.

To this end, the general illuminance around sunset in Argentina was measured using a high-spec lux meter (TES-1330A, TES Electrical Electronic Corporation, tes.com.tw; Fig. 1a). This lux meter had a range from 20.000 lux down to 0.01 lux, allowing accurate measurement of mesopic illumination, which is generally considered to be in the range from 50 to 0.1 lux [24]. The measurements were taken on 3 consecutive days and three locations in the middle of summer (29–31 January 2025) and were later repeated in one location in late summer (3 March 2025) and late autumn (8 June 2025) to assess the influence of seasons. All measurements were performed under clear, cloudless sky conditions, without mist or other atmospheric phenomena that could significantly influence ambient illumination levels. These stable weather conditions allowed for consistent and representative assessment of natural light transitions during dawn and dusk, minimising variability related to cloud cover or atmospheric scattering. No human or animal subjects were involved in the study, so ethical approval was not required.

Three locations with little artificial illumination were considered. Location A was a terrace at the roof of a high-rise building in the city of Buenos Aires (34°34′S 58°26′W; Fig. 1b). Being one of the tallest buildings in the neighbourhood, this terrace stands high above the lights emitted from other buildings, while all the lights in the terrace itself were switched off. Measurements were taken on the floor of the terrace at sunset. This location was used for repeated measurements in late summer and late autumn to ensure seasonal consistency. Location B was in the forests in the delta of the River Plate (34°22′S, 58°37′W; Fig. 1c). The measurements were done on a clear wooden pier in the river, surrounded by trees in a clear afternoon sky. The nearest house was located over 50 m from the pier, and all lights were switched off during the measurements. Location C was in the ‘*Tres de Febrero*’ park, near the first location (34°33′S, 58°25′W; Fig. 1d). The park has many large trees surrounding a lake and little artificial illumination, covering the area in deep shadows even at noon. During the measurements, all artificial lights near the trees were switched off.

Measurements were taken from 1 h before until after sunset in 10-min intervals at different times according to the season. This time window was selected to capture the transition from photopic to mesopic and early scotopic illumination, which was the primary focus of the study. After sunset, measurements continued until illuminance values approached the lower detection limit of the instrument (0.01 lux).

Before sunset, the display of the lux meter could be read directly while ensuring that the shadow of the observer did not bias the measurements. In the darkness after sunset, the hold key of the lux meter was used, and the measurements were recorded with a cell phone light while ensuring that the sensor remained unaffected. Throughout all measurements, the light sensor was positioned horizontally, facing upward toward the zenith, consistent with standard meteorological practices for environmental illuminance assessment. Horizontal positioning was selected to obtain a standardised and reproducible estimate of ambient environmental illumination, independent of observer orientation. Vertical (eye-level) measurements were not performed, as these would be highly dependent on gaze direction and relative orientation to the sun, potentially introducing substantial variability unrelated to true environmental light conditions. Moreover, the primary objective of this study was not to quantify individual ocular light exposure, but to determine the temporal dynamics of the transition from photopic to mesopic and scotopic illumination under natural outdoor conditions.

To ensure that the measurements agreed with meteorological measurements, illuminance values from the official climatological reports for Buenos Aires [25] on the dates considered were used as a reference. However, these values were only available in 1-h intervals until sunset.

## Results

Continuous lux meter recordings showed a progressive decline in ambient illumination as the Sun descended. During the early afternoon measurements, luminance decreased at approximately 5 lux/s; this rate gradually slowed with diminishing daylight, reaching <1 lux/s near sunset.

**Fig. 2:**
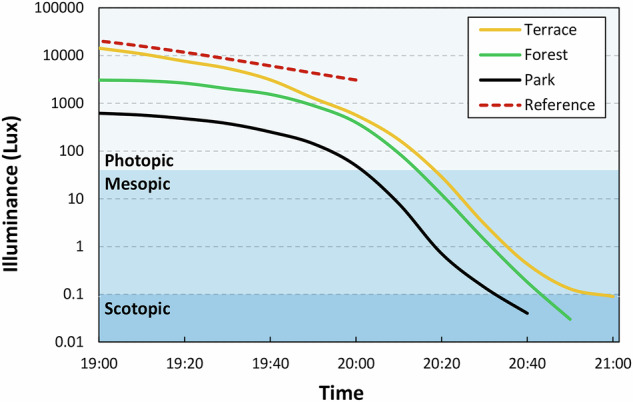
Changes in illuminance during a summer sunset (29–31 January 2025) for three locations in Buenos Aires in 10-min intervals, compared with official meteorological reference measurements in 1-h intervals.

Figure 2 shows the decay in illumination from photopic levels at 19:00 h (in summertime) to mesopic (<40 lux) or scotopic conditions (<0.1 lux) across the three measurement locations. Although the shadowy park reached mesopic illuminance 15–20 min earlier than the terrace or forest, the mesopic period in all three locations lasted about 25–30 min.

**Fig. 3:**
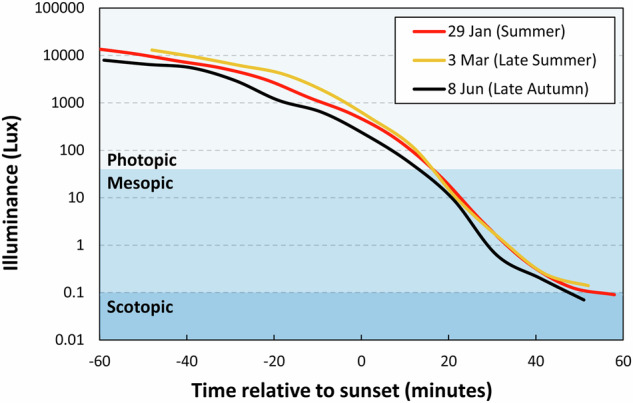
Changes in illuminance at sunset on three dates on a rooftop terrace in Buenos Aires in 10-min intervals.

Repeated measurements conducted at the terrace in late summer and late autumn showed comparable durations for the mesopic transition period (Fig. 3), indicating no observable seasonal variation in Buenos Aires under the weather conditions evaluated.

## Discussion

**Fig. 4:**
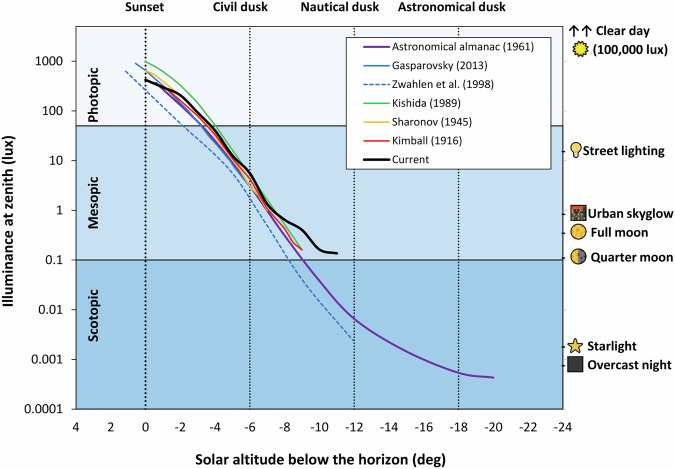
Literature data of illuminance changes around sunset across sites around the world [26–31].

**Fig. 5:**
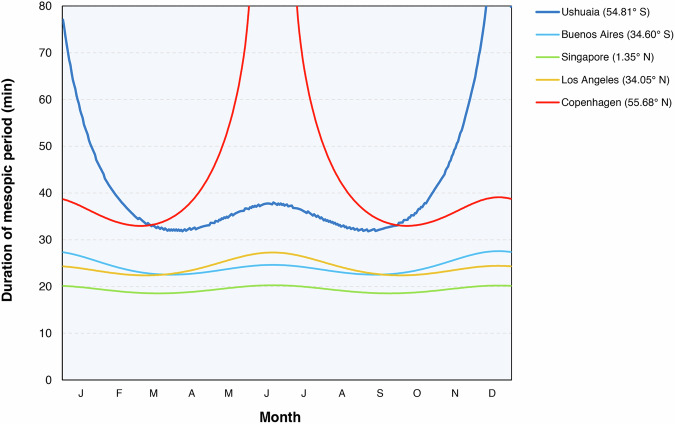
Estimated duration of the mesopic period in the evening for locations at various latitudes.The shortest durations are found at the spring and fall equinoxes, while the duration is longest at the summer solstice. The winter solstice also shows a maximum, albeit lower than the one in summer.

This study shows that in Buenos Aires, the natural mesopic illumination lasts about 25–30 min at sunset. The duration of mesopic illumination was highly consistent across the three different settings and remained stable regardless of the season. The path of the sun in the sky (ecliptic) and the obliqueness of this path when crossing the horizon at dusk or dawn predominantly depend on latitude, rather than the season of the year. A comparison with literature data [26–32] (Fig. 4) shows a general agreement between the  curves determined here and those of other regions with a latitude of roughly ±40°. This suggests that the curves presented here may be typical for the most inhabited regions of the world. This figure also shows that the mesopic period generally occurs while the Sun is between 4° and 9° below the horizon. As the solar position is readily available through online resources, it is easy to estimate the duration of the mesopic period for any other location around the world. Examples of such estimates are given in Fig. 5 for several northern and southern latitudes, showing increasing variations throughout the year, further away from the equator.

Except in regions close to the poles, the period of mesopic vision under natural circumstances is far shorter than the hours children typically spend in mesopic light environments nowadays, at least compared with the data from one seminal study by Read et al., who measured children’s light exposure with a wearable wrist device 10 years ago [22, 23]. In that study, myopic children spent less time in the dark (scotopic vision) and more time in mesopic vision compared to their emmetropic peers. The authors did not ask children about their reading habits in the dark, but in all, children spent about 4–5 h per day in mesopic vision. Preliminary data from a study conducted in India, which evaluated light exposure between 18:00 and 22:00 h in myopic and emmetropic children, showed a median evening illuminance exposure of 27 lux. Meanwhile, participants spent only 2 min (median [IQR: 0–6]) at illuminance levels above 100 lux, indicating that evening light exposure predominantly remains within the mesopic range [33]. Hence, previously published datasets using wearable sensors or digital light-recording devices, many of which have primarily focused on high outdoor illuminance thresholds, may require reanalysis to examine mesopic exposure patterns specifically [20, 21]. A seminal study just released has shown that mesopic light at night exposure of 1–10 lux is related to experimental and human myopia, showing how important this aspect is [34].

Ambient light is a major environmental factor influencing myopia development and progression [18, 35, 36]. Additionally, spending time in mesopic light levels was associated with more myopia progression in children [22] and animal models [37], while spending the last hours before sleep in scotopic light levels was found to be protective against myopia progression [22]. Recent cross-sectional Argentinian studies have shown that reading at night in mesopic light conditions may relate to myopia prevalence [38, 39]. These outcomes are consistent with the results from a mouse model, where myopic shifts induced by negative lenses were more pronounced when the animals were reared in mesopic illumination compared to photopic or scotopic light levels [37]. Furthermore, exposing human eyes to light levels <0.1 lux for 1 h leads to choroidal thinning [40], where the choroid acts as a mediator between the retina and the sclera. The choroid helps to modulate eye growth by becoming thicker when there is a STOP signal from the retina for ocular growth and thinner when there is a GROW signal. This could indicate that staying in mesopic light may produce GROW signals, as is suggested by a recent study in humans [41].

Schaeffel’s research group discovered that reading black text on a white background leads to the opposite choroidal thickness response produced by reading white text in a dark background in human eyes [42], suggesting that choroidal movements are part of the signals across the tunics of the eye from the retina to the sclera [43]. The same authors also showed that the choroids of myopes are less responsive than those of emmetropes when a stop signal in the form of positive lenses was applied [44] and that blurring the pixels of different colour channels in movies produces different choroidal responses, such as thicker choroids in myopic eyes in response to blurring the blue channel [45]. This suggests that the eye computes the relative contrast sensitivity perceived by the L, M and S-cones [46] to adjust its growth rate [10]. However, this may not be the only clue that the eye uses for detecting the image plane [47].

Besides illumination, outdoor environments present higher luminance, as well as a broader range in luminance and spatial frequencies compared with indoor environments [48]. In high luminance, the ON pathways show larger responses than the OFF pathways [12, 49, 50]. Meanwhile, when walking outside, the blinking rate, pupil size and the vestibular reflexes that reduce the residual retinal motion are driven mostly by ON pathways in the electroretinogram [51]. This presents a plausible advantage of the outdoor environment for driving ON pathways [51]. The ON-OFF contrast balance in the retina is of particular importance as it is believed that only the ON circuits can stimulate dopamine release, the main factor responsible for the STOP signal in ocular growth. It is also known that dopaminergic amacrine cells and intrinsically photosensitive retinal ganglion cells, related to the signalling of retinal circadian rhythms, are also involved in ocular growth signalling [12]. Therefore, a disturbance in the delicate balance of the ON/OFF contrast equilibrium when spending most of the time indoors could explain how the retinal pathways could lead to myopia onset [12]. This balance is very sensitive to changes in ambient illumination, as eyes tested in low luminance will see a smaller response in the ON pathways on an electroretinogram [49]. This could explain why spending a longer time in darkness just before sleep (i.e., <0.001 lux), which only stimulates the rod photoreceptors and the ON pathways, may be protective against myopia [22, 37]. Interestingly, most experiments on ON/OFF contrast or blurring colour channels have been performed in mesopic illumination, looking at screens at a 2-m distance inside a dark room [44, 45, 52]. The mesopic illumination in these studies may have influenced the results, as well as the fact that some experiments were done at a near distance, while others looked at a far distance [53]. It is also unclear what the most appropriate times are for outdoor exposure to prevent myopia, or what the interactions of the relative periods of outdoor exposure and indoor illumination exposure are throughout the day in humans [36]. A recent myopia model has been developed in guinea pigs growing with unrestricted vision, just by altering the number of hours per day that the animals are exposed to mesopic vision [54]. All of this shows that further research is necessary to further explore the role of mesopic illumination in myopia development.

Several studies also suggested that shorter sleep duration or dim lights at night could lead to a greater myopia risk [18, 33, 34, 55]. These studies, however, did not consider that many participants may have spent several hours under mesopic light levels watching content on electronic devices before sleeping. Consequently, it may not be the short duration of the sleep, but rather the longer duration of mesopic vision before sleeping that affects myopia progression. One remaining question is whether and how this extensive screen use leads to faster eye growth and myopia development.

Although exposure to low ambient light has been shown to induce myopia in chickens over 90 days with unrestricted vision [56], this effect was not observed in monkeys reared under mesopic light conditions [57]. Instead, studies in which monkeys were reared with negative or positive lenses in one eye found that low ambient lighting levels reduce the efficacy of the vision-dependent mechanisms that regulate refractive development [58]. While time outdoors has been shown to be protective against myopia onset, evidence that outdoor exposure can slow myopia progression remains limited [18]. In particular, it has not been well established whether light intensity, spectrum composition or other cofactors are key drivers of this protective effect. In fact, the observation that classrooms with walls and ceilings painted with natural scenes were associated with slower myopia progression in children [59, 60] shows how little is still known about the role of light in ocular growth. As such, quantifying mesopic light duration for myopia research represents an important area for further investigation.

Several limitations of this study should be considered when interpreting the findings. First, the research was conducted in a specific geographic area (Argentina), which may not represent mesopic light conditions fully across different regions with varying climates, landscapes and vegetation types. Additionally, measurements were taken in one region of the world, potentially limiting the understanding of mesopic light duration and transitions in different latitudes, where daylight duration and atmospheric conditions may differ significantly. In fact, the angle between the sun’s path across the sky and the horizon depends on latitude, and, in more extreme locations like the Arctic, the duration of mesopic illumination lasts longer. As three locations at one latitude cannot encompass the full range of natural environments, a larger sample size will be needed, including diverse urban-rural gradients, to provide a more comprehensive understanding of mesopic light dynamics [61]. However, Fig. 5 suggests that the time that the Sun spends between 4° and 9° below the horizon can help estimate the duration of the mesopic period. Furthermore, while the light sensor utilised has a low sensitivity threshold, environmental interferences, such as nearby artificial lighting or physical obstacles, could affect the accuracy of the recorded light levels. The temporary absence of lighting in the park location represents an unusual circumstance that may not be replicable in future studies or other settings. This raises questions about how representative these data are compared to more typical ‘natural’ environments, such as the Delta Forest site, where artificial light pollution is not present. These limitations (restricted number of measurement locations, the lack of seasonal measurements and the absence of artificial light sources) underscore the need for further research to validate and expand these findings in various contexts and under differing environmental conditions, ultimately enhancing our understanding of mesopic light conditions and their implications in myopia development.

In conclusion, this study provides objective field-based data on the characteristics and temporal dynamics of mesopic light conditions in natural outdoor environments. Although the onset of mesopic illumination varied slightly between locations, its duration in Buenos Aires remained consistent at approximately 25–30 min during summertime. Importantly, this study did not investigate myopia status, refractive development or the interaction between mesopic illumination and myopia progression. The objective was solely to characterise the duration of mesopic light exposure under natural environmental conditions. By quantifying this previously underreported environmental parameter, the present findings establish a baseline reference for future research. These data may inform comparative analyses of indoor mesopic exposure and support prospective studies using wearable light sensors to explore potential associations between mesopic illumination patterns and refractive development. Further investigations across different geographic regions, environmental settings and seasons are warranted. Integrating environmental measurements with data on children’s actual time spent under mesopic conditions in urban environments will be essential to understand real-world exposure patterns better.

## Data Availability

No datasets were generated or analysed during the current study.
